# Effectiveness of M-Health Interventions to Improve Medication Adherence in People with Schizophrenia Spectrum Disorder: A Systematic Review

**DOI:** 10.3390/nursrep16090303

**Published:** 2026-08-26

**Authors:** Worku Animaw Temesgen, Yuen Yee Lai, Ho Nam Suen, Wai Yan Chan, Pui Tik Yau, Wai Tong Chien, Yuen Yu Chong

**Affiliations:** 1 College of Medicine and Health Sciences, Bahir Dar University, Bahir Dar P.O. Box 79, Ethiopia; 2 The Nethersole School of Nursing, Faculty of Medicine, The Chinese University of Hong Kong, Hong Kong SAR, Chinajamyau@cuhk.edu.hk (P.T.Y.); wtchien@cuhk.edu.hk (W.T.C.)

**Keywords:** medication adherence, mobile health, schizophrenia, psychosis, mental health, systematic review

## Abstract

**Background**: Mobile health interventions offer a potential solution to adherence challenges, yet evidence regarding their collective efficacy in schizophrenia spectrum disorders has not been formally synthesized. This systematic review evaluates the impact of mobile health (mHealth) interventions on medication adherence as a primary outcome and on daily functioning and psychotic symptoms as secondary outcomes in individuals with schizophrenia spectrum disorders. **Methods**: Using the Population, Intervention, Comparison, Outcome (PICO) framework, a systematic search was conducted across multiple databases to identify relevant randomized controlled trials (RCTs) evaluating mHealth strategies for medication adherence in adults with schizophrenia spectrum disorders. The PubMed, CINAHL, PsycINFO, EMBASE, and JBI databases were searched from inception until 24 February 2026, using combinations of search terms such as “Schizo” OR “Psychos” AND “mHealth” OR “Digital Health” AND “Medication Adherence”. Data extraction was conducted using a standardized data extraction table and narratively synthesized. This review adheres to the Preferred Reporting Items for Systematic Reviews and Meta-Analyses (PRISMA) guidelines, ensuring structured and comprehensive reporting of the findings. **Results**: Fourteen randomized controlled trials (RCTs) with 1717 participants were included in this review. Four studies evaluated text messaging interventions, four employed phone call interventions, three used electronic medication monitoring systems, and three used mobile applications. Nine of the fourteen studies reported statistically significant improvements in medication adherence. For secondary outcomes, the results were highly inconsistent: only three studies demonstrated significant reductions in psychotic symptoms, and none showed benefits for daily functioning. While various theoretical frameworks, such as the Health Belief Model and Cognitive Behavioral Therapy and intervention modalities, were utilized, the overall evidence was limited by high clinical heterogeneity and a lack of robust long-term data. **Conclusions**: mHealth interventions, particularly text messaging and mobile applications, demonstrate clear potential to improve medication adherence in individuals with schizophrenia spectrum disorders. Given the high heterogeneity and lack of long-term evidence, future research should prioritize standardized outcome measurements, rigorous designs, and extended follow-up periods to confirm clinical utility.

## 1. Introduction

Schizophrenia is a severe mental illness leading to substantial healthcare burdens and societal impacts. According to the World Health Organization, schizophrenia affects approximately 23 million people worldwide (1 in 345; 0.29%), or 1 in 233 among adults (0.43%), and ranks among the leading causes of years lived with disability globally [1]. Psychotic symptoms of schizophrenia may lead to adverse consequences such as suicide, violent behavior, and impaired cognitive and social functioning if left untreated. Long-term antipsychotic medication emerges as the first-line treatment for reducing and controlling psychotic symptoms by stabilizing neurotransmitter activity and thus fostering optimal psychosocial functioning [2].

Medication adherence is the extent to which an individual follows the medication prescription, including the correct initiation, dosage, route, and duration as agreed with their healthcare provider [3]. However, non-adherence to antipsychotic medication among patients with schizophrenia stands out as a significant barrier to effective treatment. According to a recent systematic review, a global medication non-adherence rate of 56% was observed among patients with schizophrenia [4]. Several factors contribute to non-adherence in schizophrenia treatment, including lack of insight, adverse medication effects, the duration and complexity of the treatment regimen, economic barriers, social stigma, patient–provider relationship quality, and logistical challenges [5,6,7]. The escalating trend in medication non-adherence among patients with schizophrenia poses substantial risks of symptom relapse and a higher risk of attempted suicide [8,9].

Diverse psychosocial interventions, including Motivational Interviewing (MI), Adherence Therapy (AT), Family Therapy, medication regimen management, financial incentives, and compliance-enhancing strategies, are utilized to enhance medication adherence in this population [10,11,12]. Nevertheless, numerous studies have indicated limitations in the effectiveness of these interventions for improving medication adherence, particularly among patients with schizophrenia [13,14]. Moreover, the traditional face-to-face delivery methods present practical challenges and can lead to participant disengagement, thereby diminishing the efficacy of interventions aimed at promoting medication adherence [15].

Mobile health (mHealth) interventions, utilizing devices such as phones and personal digital assistants (PDAs), have emerged as promising tools to enhance medication adherence [5]. The scope of mHealth encompasses modern smartphone applications, short message services (SMS), and telephone voice calls. The inclusion of voice calls is clinically justified given their widespread accessibility, low technical barrier, and established utility in delivering real-time clinical support to individuals with severe mental illness who may face digital-literacy or connectivity constraints [16]. Leveraging advanced technology, mHealth interventions offer a wide array of options and online delivery platforms, significantly increasing accessibility and flexibility for patients to engage with therapeutic strategies [17].

Previous systematic reviews and meta-analyses have reported heterogeneous findings regarding the efficacy of mHealth interventions in improving medication adherence. Although several syntheses have indicated promising effects across general populations and individuals with severe mental illness [5,18,19], a recent meta-analysis of broader digital health interventions in schizophrenia spectrum disorders found no significant overall benefit. However, potential advantages were observed for human-supported modalities [20]. These divergent findings underscore persistent uncertainties and highlight the need for targeted evidence synthesis. While prior reviews have mainly merged broader digital health technologies and heterogeneous populations, comprehensive systematic reviews focusing exclusively on the intersection of mHealth modalities and medication adherence in schizophrenia spectrum disorders remain limited. Therefore, a systematic review is essential to identify and assess the extant evidence on the efficacy of mHealth interventions in enhancing medication adherence among this demographic. By synthesizing this evidence, the review aims to inform the development of innovative interventions and strategies, ultimately improving patient outcomes and healthcare delivery for individuals with schizophrenia.

## 2. Methods

This systematic review aimed to evaluate the effectiveness of mobile health (mHealth) interventions in improving medication adherence (primary outcome), daily functioning, and psychotic symptoms (secondary outcomes) in individuals with schizophrenia spectrum disorders. The review followed a protocol prospectively registered in PROSPERO (registration number: CRD42024577023) and adhered to the Preferred Reporting Items for Systematic Reviews and Meta-Analyses (PRISMA) 2020 guidelines [21].

### 2.1. Search Strategy

The research question was formulated using the PICO (Population, Intervention, Control/Comparison, Outcomes) framework. Relevant keywords, including ‘Schizophrenia,’ ‘Psychosis,’ ‘Medication Adherence,’ ‘mHealth,’ ‘Device Monitoring,’ ‘Mobile Texting,’ ‘Technology,’ ‘Smartphone,’ and ‘Non-adherence,’ were selected in alignment with the PICO criteria (see Supplementary File S1). A comprehensive literature search was conducted across several databases, including PubMed, CINAHL, PsycINFO, EMBASE, and JBI COnNect+ (Joanna Briggs Institute EBP Database), from their inception until 24 February 2026. The search strategy, detailed in Supplementary File S2, was developed in collaboration with a medical librarian to ensure the identification of relevant studies.

### 2.2. Eligibility Criteria

Eligible studies were required to employ a randomized controlled trial (RCT) design to examine mHealth interventions in adult patients (aged 18 years or older) diagnosed with schizophrenia spectrum disorders (such as schizoaffective disorder or schizophrenia-subtype disorders) according to established diagnostic frameworks, including the International Classification of Diseases (ICD) or the *Diagnostic and Statistical Manual of Mental Disorders* (DSM) criteria. Diagnoses must have been made by psychiatrists in clinical settings, with no restrictions regarding the duration of the illness. Interventions must have incorporated mHealth components delivered via mobile devices, such as mobile phones, patient monitoring devices, and personal digital assistants (PDAs). Eligible delivery modes included text messaging, smartphone applications, electronic medication monitors, and telephone calls. Given the anticipated heterogeneity of the studies, no limitations were imposed on the types of control conditions, which could include active controls, usual care (e.g., psychoeducation, supportive counseling, other therapies), or waitlist controls. Included studies were required to report medication adherence as an outcome. To maintain methodological quality, eligibility was limited to full-text, peer-reviewed studies published in English. Consequently, study protocols, gray literature, and articles without accessible full texts were excluded.

### 2.3. Study Selection and Data Extraction

EndNote 20 was initially used to remove duplicate articles. Subsequently, three independent reviewers (YYL, WAT, and HNS) screened the titles and abstracts, and then assessed the full texts of potentially eligible studies against predefined criteria. Data extraction was performed using a standardized form to record key information, including author, publication year, study setting, participant demographics (age, gender, diagnosis), sample size, intervention types, delivery methods, intervention duration, control groups, outcomes, and measurement tools. Any discrepancies were resolved by a third reviewer (YYC).

### 2.4. Data Synthesis and Analysis

A systematic narrative synthesis approach was utilized to analyze and integrate the findings from the included randomized controlled trials. Due to the profound clinical and methodological heterogeneity across the studies, a quantitative meta-analysis was deemed inappropriate and was precluded. These methodological and clinical variations prevented statistical pooling and included substantial disparities in intervention modalities, highly variable follow-up durations, and incompatible outcome metrics for the primary outcome. Specifically, medication adherence measurement tools ranged from subjective self-reports and objective electronic monitoring to biochemical verification via serum and urine bioavailability, thereby targeting fundamentally different constructs, including both actual adherence behaviors and psychological attitudes toward medication. Extracted data were synthesized and organized by type of intervention, effectiveness of primary and secondary outcomes, and user engagement and acceptability. The efficacy of the interventions was evaluated using the specific follow-up time points reported in each study, with findings compared narratively across intervention types.

### 2.5. Methodological Quality Assessment

The quality of each trial was evaluated using the Effective Public Health Practice Project (EPHPP) Quality Assessment Tool for Quantitative Studies [22]. The assessment covered five domains: selection bias, study design, confounders, data collection methods, and withdrawals/dropouts. The blinding domain of the EPHPP tool was excluded from individual study scoring due to the practical challenges of blinding in digital psychosocial interventions, where participants are inherently aware of the delivery modality [23]. Instead, this risk of bias is addressed holistically as a structural limitation of the reviewed literature. Each domain was rated as strong, moderate, or weak. A global rating was then assigned: ‘strong’ if no domains were weak, ‘moderate’ if one domain was weak, and ‘weak’ if two or more domains were weak.

## 3. Results

### 3.1. Study Selection

**Figure 1:**
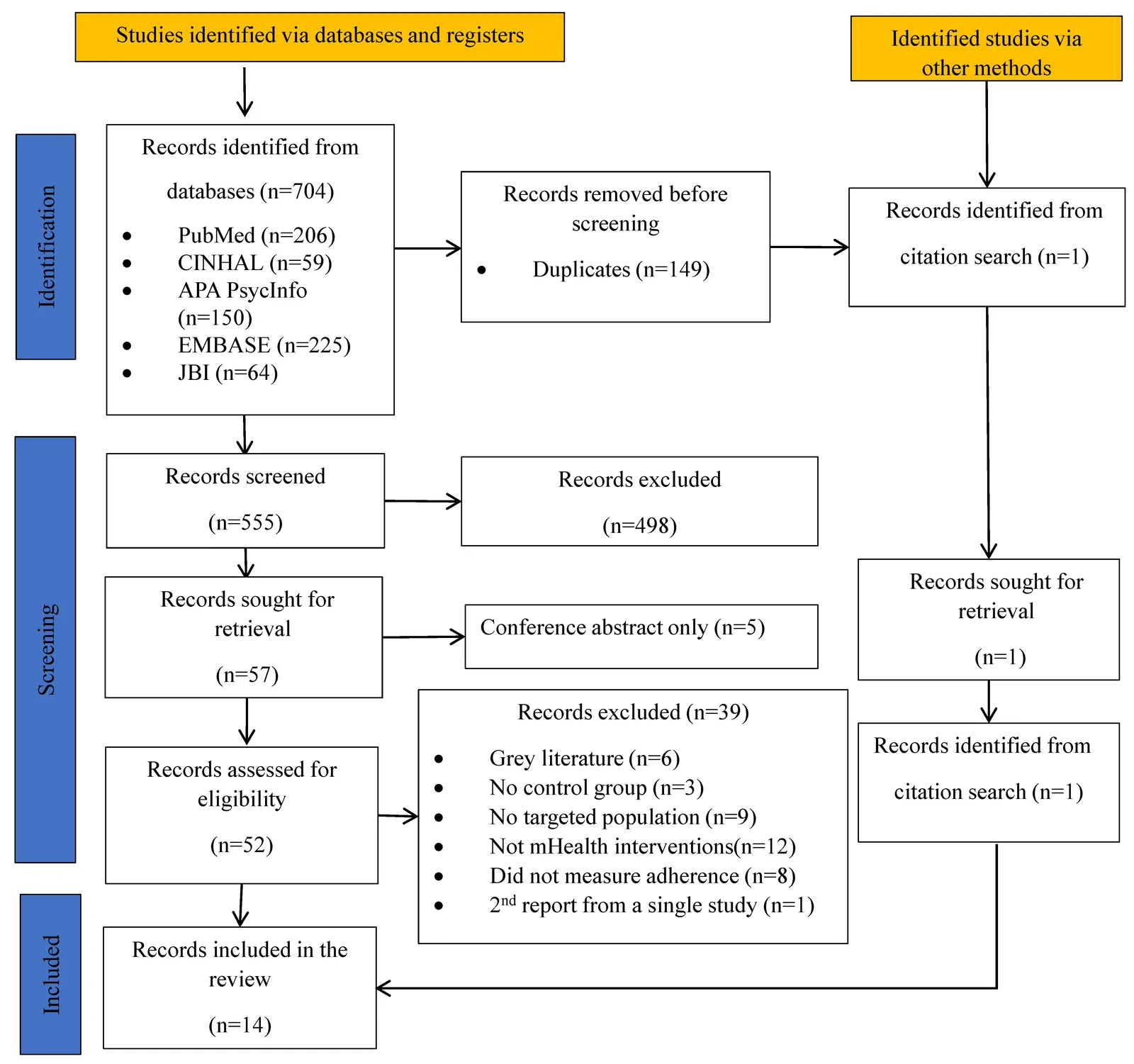
PRISMA flow diagram showing study selection process.

From an initial pool of 704 records across five databases, 149 duplicates were removed, leaving 555 for title and abstract screening. Following the exclusion of 498 records, 57 advanced to full-text review. Of these, 44 were again excluded for the following reasons: conference abstracts only, gray literature, lack of control group, ineligible target population, absence of mHealth components, or failure to measure medication adherence. Additionally, one report [24] was excluded because a more comprehensive publication from the same trial [25] was already incorporated. Citation searching identified one additional eligible article. Finally, 14 independent randomized controlled trials (RCTs) met all the inclusion criteria and were reviewed in this study. Figure 1 shows the PRISMA flow chart of study selection.

### 3.2. Characteristics of the Included Studies

Table 1 outlines the characteristics of the included studies. Of the fourteen included RCTs, twelve were two-arm designs, and two were three-arm designs. Of the fourteen included studies, nine were conducted in Western countries, four in China, and one in Turkey. Half of the interventions occurred in community settings, with four in outpatient settings, two in community and outpatient settings, and one in an inpatient setting.

### 3.3. Characteristics of Participants

The included studies encompassed 1717 participants diagnosed with schizophrenia spectrum disorders, with sample sizes ranging from 29 to 278 per trial. The gender distribution was nearly equal across studies. The mean age of participants in each study varied from 30.8 to 52 years, and the duration of illness ranged from 10 to 18.4 years. All participants were prescribed at least one oral antipsychotic medication.

### 3.4. Characteristics of the mHealth Interventions

Table 2 provides a detailed summary of the findings from the studies included in this review, which employed different intervention modalities to enhance medication adherence among individuals with schizophrenia spectrum disorder.

Mobile applications: Three most recent studies, two in China [25,26] and one in Canada [27], adopted mobile application interventions either alone or integrated with other modalities, demonstrating a shift toward multi-functional technology and approaches. Zhou et al. [26] utilized a hybrid digital medication system that combines physical smart-monitor hardware with a mobile app. The device issues voice reminders and tracks dose-taking, while the integrated app provides real-time notifications to both caregivers and healthcare workers. Liu et al. [25] adopted a narrative-based smartphone application that uses gamification and role-playing simulations rooted in Cognitive Behavioral Therapy (CBT) to enhance engagement. Similarly, Kidd et al. [27] employed a comprehensive mHealth platform, a mobile application named A4i, that offers individualized scheduling, symptom tracking, a CBT-informed resource library, and a moderated peer-to-peer social feed. These studies mark a shift from traditional to integrated digital methods and combine active monitoring, psychological intervention, and social support.

Text messaging interventions: Four studies investigated the efficacy of text messaging interventions for improving medication adherence in individuals with schizophrenia spectrum disorders. These included daily automated reminders over periods ranging from 3 to 6 months. Three of these studies implemented a two-way text messaging system that facilitated interactive communication between participants and researchers. This enhanced health education and enabled timely referrals for managing medication adherence or symptom relapses. Half of these studies also involved family members in monitoring patient progress to strengthen the support network [29,30,31]. In contrast, Montes et al. [28] utilized a one-way text messaging strategy, delivering medication reminders twice daily. The study authors reported that these interventions incorporated elements of psychoeducation and positive reinforcement; two studies stated that the Health Belief Model [39] and/or Behavioral Learning Theory [40] informed their design and intervention development.

Phone call approaches: Four studies used manualized phone calls provided weekly over two to four months to remind participants about medication and discuss adherence challenges [32,33,34,35]. These calls, conducted by nurses, included guidance on problem-solving strategies [33], with emphasis on behavioral reinforcement through reminders and feedback.

Electronic medication monitors: Three studies utilized electronic medication monitoring systems, such as the Medication Event Monitoring System (MEMS) or Med-eMonitor (MM), to track medication adherence in real-time over periods ranging from 2 to 9 months. Daily data collected from these devices were transmitted to web-based platforms, enabling clinical teams to conduct timely follow-up on adherence issues [36,38]. Additionally, Kozuki et al. [37] combined visual feedback therapy with electronic monitoring by reviewing adherence data biweekly with participants for 3 months, emphasizing behavioral reinforcement through feedback.

### 3.5. Quality of the Included Studies

The results of the quality appraisal using the EPHPP are summarized in Supplementary File S3. All studies were characterized as RCTs, suggesting robust study designs. Regarding the methodological quality score, half of the included studies (*n* = 7) received a ‘strong’ rating, while the remaining studies were classified as ‘moderate’ (*n* = 4) and ‘weak’ (*n* = 3). Twelve of these studies exhibited potential selection bias. Eight of these studies received a ‘moderate’ rating because they utilized systematic recruitment strategies. For example, they conducted comprehensive sampling by screening all potential subjects within a designated timeframe at outpatient clinics or healthcare centers. The participation rates in these studies ranged from 60% to 87% [25,26,27,30,33,35,36,38]. The remaining three studies were assessed as ‘weak’ due to their use of recruitment methods such as selection of study sites, self-referral, or chart review in outpatient settings [28,32,37]. The two studies by Beebe et al. [33,34] reported a low participation rate, highlighting significant concerns regarding selection bias that could potentially limit the generalizability of the findings. Five studies [25,26,33,34,37] lacked details regarding the control of confounders before the intervention or in the data analysis, resulting in a ‘weak’ rating in this EPHPP domain. However, all studies employed valid and reliable measures for assessing the primary outcome of medication adherence, indicating robust data collection methods. Two studies were rated as ‘weak’ in the withdrawal and dropout domain due to high attrition rates: 24% and 56.8% for Beebe et al. [33] and Beebe et al. [34], respectively. Other studies generally reported follow-up rates above 80%, reflecting strong retention.

### 3.6. Instrument Used and Data Collection Methods

The included studies assessed a range of outcomes, such as medication adherence (*n* = 14), symptoms of schizophrenia (*n* = 10), daily functioning (*n* = 3), institutionalization (*n* = 2), insight into illness (*n* = 1), and quality of life (*n* = 2), with data collection points spanning from baseline up to 18 months post-intervention. Medication adherence was measured using various methods, including self-reported questionnaires (*n* = 7) such as the Medication Adherence Rating Scale (MARS), Morisky Adherence Questionnaire (MAQ), Brief Adherence Rating Scale (BARS), Drug Attitude Inventory—10 (DAI-10), and Medication Adherence Self-Efficacy Scale (MASES) [41,42,43,44,45]; pill counts (*n* = 3); electronic monitoring devices (*n* = 4) like MEMS, or Medication Event Monitoring Systems (MEMSs); and vital statistics (*n* = 1) involving blood plasma and urine drug concentrations. Symptoms of schizophrenia were evaluated using rating scales such as the Positive and Negative Syndrome Scale (PANSS), Clinical Global Impression Scale—Schizophrenia (CGI-SCH), and Brief Psychiatric Rating Scale (BPRS) [46,47,48]. Daily functioning was assessed through instruments such as the World Health Organization Disability Assessment Schedule 2.0 (WHODAS) and Social and Occupational Functioning Assessment (SOFAS) [49,50]. Additionally, Liu et al. [25] assessed social functioning using the Social Disability Screening Schedule (SDSS).

### 3.7. Intervention Engagement and Acceptability

Across the randomized controlled trial (RCT) studies reviewed in this report, which were conducted in China, the USA, Canada, Spain, the UK, and Turkey, mHealth interventions to improve medication adherence have demonstrated feasibility and acceptability among individuals with schizophrenia spectrum disorders in both males and females, with mean ages ranging between 30 and 50. Recruitment success was evident in large-scale community studies, such as those by Cai et al. [31], Zhou et al. [26], and Xu et al. [29]. These studies successfully enrolled and randomized over 200 participants each. Smaller pilot studies [30,33,37] established the feasibility of mHealth and telephone-based interventions in smaller samples (*n* < 50). Engagement and retention rates were consistently high across modalities, with significant treatment effects and sustained participant use over periods of up to 12 months [26,38]. The inclusion of caregivers and lay health supporters in digital platforms [26,31,36] appeared to bolster engagement. Participants in a mobile application intervention reported high satisfaction and perceived utility in qualitative follow-ups, particularly regarding medication and goal reminders [27]. Generally, these findings suggest that while clinical symptom improvement varies, the structural implementation of these interventions is robustly feasible across diverse outpatient and community rehabilitation settings.

### 3.8. Intervention Effect on Medication Adherence

All fourteen included studies evaluated medication adherence as the primary outcome. Overall, most trials (*n* = 10) reported improvement in medication adherence in the intervention group compared with the control group; however, the magnitude, statistical significance, and durability of effects varied across intervention modalities and outcome measures.

Mobile applications: Findings for mobile application interventions were promising but mixed. Two studies reported significant post-intervention improvements. Liu et al. [25] found that the gamified app significantly improved both medication adherence and medication attitude. Scores on the Medication Adherence (MMA) Scale increased from 6.9 to 7.7 in the intervention group and were significantly higher than those in the control group at post-intervention (*p* = 0.02). Zhou et al. [26] reported the largest and most sustained adherence effect among the included studies. In this trial, the hybrid digital medication system, which combined a hardware monitor with a mobile application, yielded a 12-month mean adherence rate of 77.7% in the intervention group compared with 23.4% in the control group (*p* < 0.001). In addition, Medication Adherence Rating Scale (MARS) scores improved significantly over time, increasing from 4.74 at baseline to 7.68 at 12 months (*p* < 0.0001). In contrast, Kidd et al. [27] found no statistically significant between-group difference in adherence over 6 months. Notably, adherence in the intervention group decreased slightly from 85.0% to 80.0% (*p* = 0.68). However, qualitative findings indicated that participants valued the application’s reminder functions for medication-taking and goal attainment. Taken together, these findings suggest that app-based interventions may improve adherence, particularly when they combine monitoring with active behavioral or social support, although the evidence remains limited.

Text messaging interventions: Among the four studies using text messaging interventions, two reported statistically significant improvements in medication adherence immediately after the intervention [28,29], with reported *p*-values ranging from 0.03 to 0.02 and effect sizes (Cohen’s d values) of at least 0.38. In addition, sustained benefits were observed in follow-up assessments in some studies, including at the third month in Montes et al. [28] (*p* = 0.04) and at the 18th month in Cai et al. [31] (mean difference 0.05, although not significant (*p* = 0.11)). Cullen et al. [30] reported a favorable trend in oral medication adherence at 6 months, with adherence rates of 89% in the intervention group and 69% in the control group, although not statistically significant (*p* = 0.11). Overall, the text messaging literature suggests that this modality may improve adherence, with some evidence of maintenance beyond the active intervention period.

Phone call approaches: Phone call interventions showed some evidence of short-term benefit. Beebe et al. [32] and Uslu et al. [35] reported improved adherence during the intervention period, with *p*-values ranging from 0.001 to 0.02. However, neither study assessed post-intervention maintenance. In a six-month study, Beebe et al. [34] found that participants in the TIPS intervention group were nearly twice as likely as controls to achieve serum antipsychotic levels within the therapeutic range (*p* = 0.02). However, no significant difference was found when adherence was measured by pill count (*p*-value not reported). None of the studies using phone-based interventions evaluated the long-term sustainability of adherence gains. Thus, although weekly support calls may improve adherence while active contact is maintained, evidence regarding durability remains lacking.

Electronic medication monitors: All three studies using electronic medication monitoring systems [36,37,38] found significant improvements in medication adherence immediately post-intervention, with reported *p*-values ranging from <0.001 to 0.03. These interventions typically used real-time monitoring data to trigger clinical follow-up or provide adherence feedback to participants. However, none of these studies examined whether the observed improvements were sustained after the intervention period. Accordingly, although findings were consistently favorable in the short term, the long-term sustainability of these effects remains uncertain.

### 3.9. Intervention Effect on Daily Functioning

Five reviewed studies measured functioning [25,26,29,31,38]. Zhou et al. [26] reported a significant reduction in the Family Burden Scale (FBS) compared to the control group (13.25 vs. 19.26, *p* < 0.001). Xu et al. [29] reported a reduced loss in functioning immediately post-intervention with text messaging interventions compared with the control group, although the result was not statistically significant (*p* = 0.117; d = 0.18). Liu et al. [25] observed a statistically significant improvement within the intervention group, reducing from a mean of 2.2 to 1.1 (*p* = 0.03); however, there was no significant difference from the control group (*p* = 0.34). Three studies [27,31,38] found no significant improvement in functioning immediately post-intervention relative to the control group. Moreover, Cai et al. [31] noted no significant sustained effect of text messaging interventions on daily functioning.

### 3.10. Intervention Effect on Psychotic Symptoms

Ten of the fourteen studies examined clinical symptoms of psychosis. Among these, three studies reported significant improvements in overall clinical symptoms immediately post-intervention compared with the control group [28,30,36], with *p*-values below 0.02, 0.03, and 0.004, respectively. The interventions leading to improvement included text messaging interventions in two studies [28,30] and electronic medication monitoring in one study [36]. Additionally, Montes et al. [28] observed a sustainable reduction in negative symptoms of schizophrenia with text messaging interventions, as evidenced by a significant *p*-value of 0.03. Other studies, including those that utilized mobile application interventions, did not find significant improvement in clinical psychiatric symptoms. Liu et al. [25] observed a reduction in BPRS symptom scores from 28.4 at baseline to 21.4 in the third month; however, this was not statistically significant either within the group or compared with the control group. Similarly, Kidd et al. [27] found no significant difference in symptom scales between the treatment and control groups; the qualitative part of their study, however, suggested that patients perceived that the apps helped to manage sleep, mood, and overall motivation, which are critical precursors to symptom stability.

## 4. Discussion

This systematic review critically assessed the effectiveness of mobile health (mHealth) interventions in enhancing medication adherence and managing daily functioning and psychotic symptoms in patients with schizophrenia. This review found that the technological transition of mHealth for schizophrenia care has shifted from passive monitoring devices to an integrated interactive digital application. Early interventions relied on electronic medication monitors (MEMSs) to serve as ‘electronic witnesses’ of patient behavior [36,37]. These were followed by a period characterized by telecommunication-based approaches, including structured telephone support [32,34] and short message service (SMS) interventions [28,29]. More recent studies [25,26,27] reflect a further shift toward comprehensive mobile applications incorporating behavioral tracking, CBT-informed gamification, and real-time social or clinical connectivity. Taken together, this progression indicates that mHealth has developed from a relatively simple reminder system into a more comprehensive and potentially therapeutic digital companion.

Analyzing fourteen RCTs, the results indicated that ten trials consistently demonstrated improvements in medication adherence immediately post-intervention. However, the effects on psychotic symptoms were mixed, with improvements noted in only four of the studies assessing this outcome. Only two [25,26] of the most recent studies conducted in China using mobile applications found significant improvements in daily functioning in their intervention groups. Notably, text messaging interventions represented the most frequently studied mHealth modality, with four reviewed trials [28,29,30,31] of moderate to strong methodological quality. These studies suggest that text messaging interventions may support medication adherence and, in some cases, sustain these improvements for at least three months.

The reviewed text messaging interventions typically involved approximately three months of daily reminder messages, often supplemented with psychoeducational content, positive reinforcement, and, in some studies, family involvement [28,29,30,31]. The interventions were primarily informed by the Health Belief Model (HBM) [39] and Behavioral Learning Theory [40]. The HBM proposes that health behaviors are shaped by perceptions of illness severity, susceptibility, benefits of adherence, and barriers to action, whereas Behavioral Learning Theory emphasizes behavior change through environmental contingencies, associative learning, and reinforcement. Drawing on these frameworks, the interventions combined simple, structured reminders with psychoeducation and reinforcing feedback to address perceived barriers, emphasize the consequences of non-adherence, and strengthen routine medication-taking behavior. This theoretical alignment helps explain why text-messaging interventions demonstrated such consistent empirical success in improving adherence. Within the framework of the HBM, automated daily texts function as an immediate, highly effective motivator for action that bypasses the cognitive and memory deficits common in schizophrenia [18,51]. Furthermore, by embedding psychoeducational content within the messages, these interventions directly modified patients’ perceived barriers and elevated their ‘perceived benefits’ of continuous treatment. This theoretically grounded design likely contributed to their feasibility and potential applicability across diverse clinical settings to mitigate medication non-adherence in schizophrenia.

The prominence of text messaging interventions in mHealth strategies stems from its acceptability, cost-effectiveness, and ease of integration into clinical workflow [18,51]. Text messaging interventions facilitate prompt data input, distribution, and retrieval [52], allowing patients to access services unrestricted by time or location [53]. Compared with telephone-based intervention, text messaging may reduce administrative burden and service costs for healthcare providers [54]. Their use is additionally supported by broader evidence from other long-term conditions. Systematic reviews on HIV, diabetes, and cardiovascular disease have shown that text-based interventions can improve medication adherence [55]. However, a critical divergence emerges when comparing our findings to reviews on chronic physical health conditions. While automated text reminders show linear, highly sustainable adherence improvements in diabetes and cardiovascular care, their efficacy in schizophrenia spectrum disorders appears far more volatile and prone to rapid decay post-intervention. This divergence is likely explained by the unique neurocognitive and symptomatic trajectories associated with schizophrenia. Unlike physical conditions, cognitive impairments, motivational deficits, and acute paranoid ideation can disrupt digital engagement, meaning text-based interventions in this population require continuous, adaptive customization rather than static automation.

More recently, a study in populations with serious mental illness has also reported beneficial effects of text messaging on adherence and clinical outcomes [56]. However, this promising utility must be interpreted with caution due to systemic methodological limitations across the reviewed trials. The evidence is limited by a lack of double blinding. Furthermore, many studies were susceptible to selection bias through highly restrictive inclusion criteria, and there remains a critical absence of robust, long-term follow-up data to confirm whether these initial behavioral gains are sustained.

Phone call interventions, delivered weekly by nurses over 2–4 months, focused on medication reminders, exploration of adherence barriers, and problem-solving support [32,33,34]. These interventions emphasized behavioral reinforcement through consistent reminders and positive feedback to establish adherence routines, supplemented by cognitive behavioral problem-solving to address personal and contextual barriers, although explicit theoretical frameworks were not always stated. Electronic monitoring interventions, implemented over 2–9 months [36,37,38], similarly drew on behavioral reinforcement principles by providing real-time adherence data and timely clinical feedback, producing significant short-term improvements in adherence. However, neither phone-based nor electronic monitoring approaches evaluated post-intervention or longer-term outcomes, limiting conclusions about sustainability and suggesting possible dependence on continuous support. Taken together, these findings highlight heterogeneous theoretical articulation across mHealth modalities, with phone-based and monitoring interventions relying largely on implicit behavioral principles compared with the more explicitly model-driven text messaging interventions. This variability, alongside the predominance of short-term assessments, underscores the need for future research to employ clearer theoretical frameworks, standardize operationalization of behavioral constructs, and incorporate extended follow-up to clarify mechanisms of sustained adherence change.

Moreover, the conceptual heterogeneity embedded within mHealth warrants acknowledgment. Treating these diverse modalities as a single category obscures their vastly distinct mechanisms of action. For instance, unidirectional SMS interventions operate primarily as simple external prospective memory aids. In contrast, telephone-based support relies on real-time human therapeutic alliance and interpersonal accountability, while electronic monitoring devices such as MEMSs leverage immediate feedback loops. Emerging mobile applications represent a markedly different paradigm, utilizing complex cognitive behavioral strategies and gamified motivation to actively reshape health beliefs [57]. Future research must move beyond evaluating mHealth broadly and instead isolate how these distinct technological architectures differentially engage patients in their treatment adherence and subsequent recovery.

Individuals with SMI need to be treated with person-centered approaches. Personal, functional, and clinical recovery from SMI can be better achieved when interventions are bidirectional, flexible, and individualized. Our findings demonstrate that newer mobile applications fulfill this need by replacing static digital reminders with dynamic personalization [25,26,27]. Zhou et al. [26] demonstrated that the greatest gains in medication adherence were achieved by merging physical monitoring hardware with mobile connectivity, while Liu et al. [25] utilized high-engagement gamification and CBT-informed narrative psychology to successfully shift patient attitudes and internal social functioning, highlighting the power of immersive, individually paced technology to bring the desired change. However, maintaining persistent engagement, as seen in Kidd et al. [27], remains a critical challenge in such new autonomous intervention modalities.

All reviewed studies, with the exception of Zhou et al. [26], Uslu et al. [35], Beebe et al. [32], and Beebe et al. [34], assessed psychiatric symptoms and daily functioning as secondary outcomes. The findings indicate that while text messaging interventions may hold promise for improving clinical symptoms, their effects on daily functioning remain inconclusive. This inconsistency across secondary outcomes likely reflects both the brief duration of interventions and the low therapeutic intensity of digital reminders, neither of which may be sufficient to meaningfully alter complex psychotic symptoms. Furthermore, because primary medication adherence in most reviewed studies relied on subjective self-reports, social desirability bias may have led participants to overreport their treatment compliance, meaning true adherence may have been lower than reported, which would help explain the absence of symptomatic improvement despite reportedly high adherence. Consequently, the apparent divergence between adherence gains and stable symptoms highlights a critical need for objective adherence metrics and extended follow-up periods in future trials.

A further nuance in intervention delivery concerns the extent of human support. While some text messaging interventions were predominantly automated (e.g., unidirectional reminders in [28,30], others incorporated bidirectional communication or family involvement as human facilitators [29,31]. Phone call interventions inherently relied on direct nurse-delivered support, and electronic monitoring typically involved clinician feedback on adherence data. Mobile application interventions support human involvement, either by alerting family members and healthcare providers as in [26] or through peer-to-peer interaction as in Kidd et al. [27]. Arnautovska et al. [58] highlight the critical role of human involvement in digital mental health interventions for schizophrenia spectrum disorders, where cognitive impairments and motivational deficits may limit engagement with fully automated tools. In the present review, combined approaches integrating mHealth with human support (e.g., family-assisted text messaging, peer-to-peer interaction, and alerts to caretakers and healthcare providers) improved adherence but demonstrated neither superiority over simpler, often more automated, modalities, nor consistent benefits for secondary outcomes. This pattern suggests that judicious, tailored integration of human elements may optimize efficacy while minimizing resource demands, warranting targeted evaluation in future trials.

Non-pharmacological interventions, including mHealth, are generally perceived as safer than pharmacotherapy or invasive treatments [59]. However, in vulnerable populations such as individuals with schizophrenia, technological, psychosocial, and behavioral interventions require careful design and systematic monitoring for potential psychological harms, including adverse mental health events [51,60,61]. The included studies took steps to mitigate risks, in most cases, incorporating bidirectional communications to allow monitoring of immediate effects by recruiting clinically stable participants who provided informed consent. Nonetheless, systematic reporting of adverse events or psychological safety outcomes was limited across the trials. Furthermore, most interventions lacked user co-design and personalization, while unidirectional approaches ([25,28,30] posed risks of unmonitored undesirable responses. These observations highlight a broader gap in standardized safety assessment for digital mental health interventions and underscore the need for future trials to adopt comprehensive monitoring frameworks.

### Limitations

A key limitation of this review is the substantial clinical and methodological heterogeneity observed across the reviewed studies, which precluded meta-analysis or quantitative subgroup analysis. The substantial diversity in intervention modalities, diagnostic sample characteristics, and follow-up durations made direct statistical pooling clinically inappropriate. Most notably, outcome measurement metrics for medication adherence varied incompatibly across studies. These tools span a wide operational range, from subjective self-reports and objective electronic pill monitoring to biochemical verification via serum bioavailability, and targeted fundamentally different constructs, with some scales quantifying actual adherence/non-adherence behaviors while others measured psychological attitudes toward medication. Consequently, a narrative synthesis approach was necessitated to faithfully represent these disparate data types without introducing artificial or misleading pooled effects. While the blinding domain was excluded from the overall Effective Public Health Practice Project (EPHPP) quality assessment owing to the nature of mHealth behavioral interventions, the lack of participant and personnel blinding domains could introduce potential performance bias that should be considered when interpreting intervention efficacy. Furthermore, in this review, medication adherence was assessed only at the level of overall antipsychotic treatment adherence, without examining differences by medication type or the number of medications prescribed. Finally, the scope of this review was limited to manuscripts published in English, potentially excluding relevant findings from studies published in other languages.

## 5. Conclusions

Mobile health interventions demonstrate short-term efficacy in improving medication adherence among individuals with schizophrenia spectrum disorders, although their effects on daily functioning and psychotic symptoms remain mixed. Among the available modalities, text messaging interventions appear highly scalable and operationally efficient across diverse care settings, whereas application-based platforms offer greater opportunities for individualized and interactive support. However, drawing definitive conclusions across all modalities remains constrained by high technological heterogeneity, incompatible adherence metrics, and a lack of data on long-term sustainability. Furthermore, most existing trials have been conducted in high-income countries, limiting the generalizability of current evidence to low- and middle-income settings. Further research in more diverse populations and service contexts is therefore needed. Larger trials with longer follow-up periods, objective adherence metrics, and standardized operational definitions of intervention components are also required to determine the clinical and scientific significance, long-term sustainability, and feasibility of implementing these interventions.

## 6. Implications for Patient Care

This study supports the potential of mHealth interventions, such as mobile applications and daily text messaging, to improve medication adherence among individuals with schizophrenia spectrum disorders. Healthcare professionals may consider incorporating these interventions as complementary components within routine care settings. However, evidence regarding their impact on symptom control and functioning remains inconclusive, and findings should be interpreted with caution. Furthermore, given the current heterogeneity in intervention types and outcome measurements, stakeholders should view mHealth as a promising but still evolving complement to standard care.

## Figures and Tables

**Table 1:** Characteristics of included studies.

Study	Study Design	Study Setting	Countries	Diagnosis	Sample Size (IG: CG)	Age Mean (SD)	Gender (Male: Female)
Zhou et al., 2025 [26]	Cluster-RCT (2-arm)	Community	China	Schizophrenia or bipolar disorder	216 (108:108)	Median 52.2, IQR (42.6–58.7)	IG (45:63) CG (45:63)
Liu et al., 2025 [25]	RCT (2-arm)	Community Rehab	China	Schizophrenia	70 (35:35)	44.2 (8.06)	IG (23:12) CG (20:15)
Kidd et al., 2025 [27]	RCT (2-arm)	Outpatient and Community	Canada	Schizophrenia	91 (43:48)	30.8 (10.9)	IG (27:14) CG (27:18)
Montes et al., 2012 [28]	RCT (2-arm)	Outpatient	Spain	Schizophrenia	254 (100:154)	39.8 (10.9)	IG (65:35) CG (104:5)
Xu et al., 2019 [29]	RCT (2-arm)	Community	China	Schizophrenia	278 (139:139)	46.5 (12.7)	IG (62:77) CG (62:77)
Cullen et al., 2020 [30]	RCT (2-arm)	Community	USA	Schizophrenia/Schizoaffective	40 (28:12)	48.7 (13.7)	IG (16:12) CG (7:5)
Cai et al., 2020 [31]	RCT (2-arm)	Community	China	Schizophrenia	277 (139:138)	46.5 (12.7)	IG (62:77) CG (61:77)
Beebe et al., 2008 [32]	RCT (2-arm)	Community	USA	Schizophrenia, or other subtypes	25 (13:12)	52 (Not Mentioned)	IG (7:6) CG (8:4)
Beebe et al. 2016 [33]	RCT (2-arm)	Outpatient	USA	Schizophrenia/ Schizoaffective	140 (Not mentioned)	46.1 (12.9)	80:60
Beebe et al. 2017 [34]	RCT (2-arm)	Outpatient	USA	Schizophrenia/ Schizoaffective	105 (Not mentioned)	46.8 (12.9)	59:46
Uslu et al. 2020 [35]	RCT (2-arm)	Inpatient	Turkey	Schizophrenia	46 (22:24)	37.8 (Not Mentioned)	IG (9:13) CG (10:14)
Frangou et al., 2005 [36]	RCT (3-arm)	Community	UK	Schizophrenia	108 (Routine care: Pill counting: @HOME 36:36:36)	47.4 (10.3)	Routine care (13:23) Pill counting (7:29) @HOME (5:31)
Kozuki et al., 2006 [37]	RCT (2-arm)	Community	USA	Schizophrenia/schizoaffective	30(15:15)	46.5 (8.62)	17:13
Velligan et al., 2013 [38]	RCT (3-arm)	Community	USA	Schizophrenia/ Schizoaffective	142 (MM: PharmCAT: TAU 47:48:47)	42.52 (10.27)	MM (27:21) PharmCAT (24:23) TAU (25:22)

**Table 2:** Data extraction table and main findings of the included studies.

Author (Year)	Description of Intervention	Control	Outcome Measures/Instruments	Duration of Interventions; Assessment Timepoints	Results: -Medication Adherence -Symptoms of Schizophrenia -Level of Daily Functioning
Zhou et al., 2025 [26]	Digital medication system (medication monitor plus mobile app): The monitor stores medication, issues voice reminders for the patient, and transmits data to the cloud server for remote monitoring by healthcare workers and caregivers.	TAU: CG received standard care with the same app for adherence tracking via an online medication diary, but no digital medication monitor or caregiver support.	- Medication adherence: self-report percentage of missed doses; - Medication Adherence Rating Scale (MARS); - Family burden scale; - Visual analog scale (VAS) for feasibility.	Assessment time points: 1, 3, 6, and 12 months for medication adherence and 3, 6, and 12 months for acceptability.	Medication adherence: Self-reported medication adherence (80% adherence to prescribed doses in the past month). Mean adherence in IG vs. CG was 77.70% vs. 23.4% at the 12th month, *p* < 0.001 (not measured at baseline) MARS: IG at the 12th month was 7.68 vs. 4.47 in CG (*p* < 0.001), with a significant improvement from baseline for IG from 4.74 at baseline to 7.68 at the 12th month (*p* < 0.0001). Family Burden (FBS): At the 12th month, FBS scores in IG vs. CG were 13.25 vs. 19.26, and were significantly lower at all visits (*p* < 0.001). VAS for acceptability and feasibility demonstrated a continuous increase over the follow-up period.
Liu et al., 2025 [25]	Mobile App: Narrative-based psychoeducational intervention three times per week for 3 months, with gamified intervention model: Narrative psychology and Cognitive Behavioral Therapy (CBT); Immersive narrative gaming/roleplaying; Adaptive Cognitive Training with real-world simulations of function. Integrated Self-Management with real-time behavioral tracking such as medication.	TAU: Standard community rehabilitation, home visits, and education on medication adherence.	- Medication adherence (MMA); - Drug attitude (DAI); - Social functioning - (SDSS); - Psychiatric symptoms (PANSS, BPRS).	3-months intervention (3/week): four measurements (T0 = pre-intervention; T1 = at completion of intervention; T2 = one month after completion of the intervention; T3 = 3rd month after completing intervention/6th month after starting intervention).	Significant improvement in medication adherence (MMA): Mean change in IG 6.9 to 7.7 (*p* = 0.03) and CG 7.2 to 6.7 with *p* = 0.11 from pre-intervention to 6-month post-intervention test with significant between-group differences (*p* = 0.02). IG had higher adherence compared to CG (*p* < 0.001 at T1, T2, and T3). Medication attitude (DAI): Mean change in IG from 4.9 to 8.9 (*p* < 0.001) and in CG from 5.8 to 6.3, with *p* = 0.47, from pre-intervention to the 3-month post-intervention test, with significant between-group differences (*p* = 0.02). IG had a better medication attitude compared to CG (*p* < 0.001 at T1, T2, and T3). Clinical Symptoms (BPRS): Mean change in IG from 28.4 to 21.4 (*p* = 0.06) and in CG from 25.8 to 26.7, with *p* = 0.66, from pre-intervention to the 3-month post-intervention test, with no significant between-group differences (*p* = 0.12). Social Functioning (SDSS): Mean change in IG from 1.2 to 1.1 (*p* = 0.03) and in CG from 2.2 to 2.9, with *p* = 0.85, from pre-intervention to the 3-month post-intervention test, with no significant between-group differences (*p* = 0.34).
Kidd et al., 2025 [27]	Mobile App: An interactive smartphone-based mHealth application (A4i); Individualized wellness scheduler (medication and appointment reminders); A CBT-informed resource feed and a personal resource library; The app tracks symptoms, activities, and goal attainment daily, and suggests support accordingly; Moderated peer-to-peer feed, and clinician dashboard for tracking.	TAU	Feasibility indicators: - Recruitment; - Retention; - Satisfaction; - Safety; - Engagement. Symptoms: - PANNS; - BPRS. Quality of life: - QLS. Treatment adherence: - MOS; - BARS; - CAMH.	6 months of intervention duration. Measurement: Baseline and 6th month.	No significant differences between treatment and control groups in medication non-adherence (BARS: mean change in IG from 85.0% to 80.0% and in CG from 88.5% to 84.6%, with *p* = 0.68, from pre-intervention to 6-month post-intervention test). Symptom scales (PANSS: mean change in IG from 54 to 48 and in CG from 56.1 to 45.1, with *p* = 0.48, from pre-intervention to 6-month post-intervention test). Quality of life (QLS: mean change in IG from 77.4 to 63.2 and in CG from 79.2–70.1 to 45.1, with *p* = 0.47, from pre-intervention to 6-month post-intervention test). - Treatment adherence (MOS: mean change in IG from 25.5 to 21 and in CG from 23.2 to 23.0, with *p* = 0.7, from pre-intervention to 6-month post-intervention test). Twenty-one individuals from IG participated in a qualitative interview and reported that the intervention was useful for - Medication reminders; - Goal reminders; - Sleep and mood; - Motivation; - Recovery if recent onset.
Montes et al., 2012 [28]	Text messaging: Daily SMS reminders. Receiving daily messages at either 11 am or 2 pm. Text content: “Please remember to take your medication.”	TAU (Patients did not receive SMS messages or any other more intensive approach for increasing adherence than standard of care during the study period).	Medication adherence (P): - MAQ; - DAI-10. Clinical severity (S): - CGI-SCH-S; - CGI-SCH-DC. Insight (S): - SUMD. Health-related quality of life (S): - EQ-5D.	3 months. Baseline, 3rd and 6th months.	1a. ↑ Adherence in both groups over 3 months. Mean changes in MAQ total score: −1.0 (95% CI −1.02, −0.98) in IG, −0.7 (95% CI −0.72, −0.68) in CG; *p* = 0.02 1b. ↑ Adherence in both groups over 6 months. Mean changes in MAQ total score: −1.1 (95% CI −1.12, −1.08) in IG, −0.8 (95% CI −0.81, −0.78) in CG; *p* = 0.04 2. Clinical severity CGI-SCH-SI: Negative symptoms: Significantly reduced in the 6th month; mean changes in CGI-SCH-SI negative symptoms subscale: IG (−0.6, 95% CI −0.62, −0.58), CG (−0.3, 95% CI −0.32, −0.28), *p* = 0.03. Mean changes in the scores of the negative symptoms subscale in the 3rd month: IG 3.3 (3.10, 3.50), CG 3.5 (3.36, 3.64), *p* = 0.02. Mean changes in scores of the cognitive symptoms subscale in the 3rd month: IG 3.3 (3.12, 3.48), CG 3.6 (3.46, 3.74), *p* = 0.01. Mean changes in scores of the global symptoms subscale in the 3rd month: IG 3.2 (3.02, 3.38), CG 3.5 (3.36, 3.64), *p* = 0.012.
Xu et al., 2019 [29]	Text messaging: LEAN Program: (1) Lay health supporters; (2) e-platform (texting); (3) Award; (4) Integration of texting with the existing health system to enable collaborative care.	Waitlist control 686 programs (i.e., integrated community-based mental health care system) in China.	Medication adherence (P): - Pill counts; - DAI-10; - BARS; - Refill record. Patient symptoms (S): - CGI-SCH-SI; - CGI-SCH-DC. Functioning (S): - WHODAS.	6 months. Baseline, 6th month (Note: pill count was not performed at the baseline).	1. Medication adherence: higher in IG than CG in the 6th month (i.e., post-intervention) (adj. mean difference 0.12 [95% CI 0.03–0.22], *p* = 0.013); no significant differences were found in other measurement tools. 2. No significant improvement in the severity of symptoms in either group. 3. Slightly less loss of functioning in IG than in CG, though the difference was not statistically significant (mean WHODAS score 0.12 in IG and 0.15 in CG (adj. mean Difference −0.03 [95% CI −0.07–0.01]; *p* = 0.117; Cohen’s d effect size 0.18).
Cullen et al., 2020 [30]	Text messaging: Texting for Relapse Prevention Program (T4RP). Daily text message about: (1) Early warning symptoms; (2) Medication; (3) Adherence/coping with side effects; (4) Presence of symptom: receive coping skills; (5) Suggestions specific to that symptom; (6) Absence of symptom: receive supportive text messages.	TAU	Symptoms (P) - PANSS. Institutionalization (P): - Information from providers. Medication adherence (S): - BARS.	6 months. Baseline, 3rd and 6th months.	1. Positive symptoms were significantly lower in the PANSS mean score in the 6th month (mean: 14.3 (SD 3.7) in CG; 11.4 (SD 3.5) in IG, *p* = 0.03). 2. Oral medication adherence improved in both the 3rd and 6th months, but not significantly. Mean adherence rate in the 3rd month: 85% (SD 11%) in CG; 96% (SD 27%) in IG, *p* = 0.18. Mean adherence rate in 6th month: 69% (SD 9%) in CG; 89% (SD 25%) in IG, *p* = 0.11
Cai et al., 2020 [31]	Text messaging: LEAN Program: (1) Lay health supporters (often a designated family member assigned to monitor patient medication, side effects, relapses, and urgent care); (2) e-platform (texting system for medication reminders, health education, and relapse monitoring); (3) Award (token gifts to encourage behavioral improvement); (4) Integration of texting with the existing health system to enable collaborative care.	Waitlist control 686 programs (i.e., integrated community-based mental health care system) in China.	Medication adherence (P): - Pill counts; - Refill record. Patient symptoms (S): - CGI-SCH-SI; - CGI-SCH-DC. Functioning (S): - WHODAS.	6 months. 18 months after suspension of intervention.	1. Medication adherence between IG and CG has become insignificant, mainly due to increased adherence in CG. Mean 0.62, SD 0.30 in IG and mean 0.58, SD 0.45 in CG; adj. mean difference 0.05, [95% CI −0.06–0.16]; *p* = 0.41; Cohen’s d effect size = 0.11. 2. No significant improvement in the severity of symptoms in either group. CGI-SCH-SI with scores between groups being 2.50 (SD 1.08) and 2.58 (SD 1.17; adj. mean difference −0.02, [95% CI −0.29–0.24]; *p* = 0.86; Cohen’s d effect size = 0.07). CGI-SCH-DC with scores between groups being 3.16 (SD 1.13) and 3.10 (SD 1.20; adj. mean difference 0.05, [95% CI −0.23–0.34]; *p* = 0.72; Cohen’s d effect size = 0.05). 3. No significant improvement in functioning in either group. WHODAS mean scores of 0.16 (SD 0.17) and 0.20 (SD 0.22; adj. mean difference −0.03, 95% CI −0.08–0.02; *p* = 0.19; Cohen’s d effect size = 0.20.
Beebe et al., 2008 [32]	Phone calls: Telephone Intervention Problem Solving (TIPS). Weekly telephone intervention by nurses to: (1) Support problem-solving; (2) Offer coping alternatives; (3) Provide reminders to use alternatives; (4) Assess effectiveness.	TAU	Medication adherence (P): - Pill count.	3 months. 3rd month.	1. Better adherence was reported in IG in the 3rd month when compared to CG. Least-squares means indicated an average psychiatric medication adherence of 80% (95% CI = 67–88%) for IG, and 61% (95% CI = 47–78%) for CG. Significant main effect in improving psychiatric medication adherence over time for IG (F (1, 20) = 5.47, *p* = 0.0298).
Beebe et al. 2016 [33]	Phone calls: Telephone Intervention Problem Solving (TIPS). Weekly telephone intervention by nurses to: (1) Support problem-solving; (2) Offer coping alternatives; (3) Provide reminders; (4) Assess effectiveness.	TAU (TIPS via landline telephone; participants in CG had no contact with the TIPS provider while hospitalized, but IG did).	Medication adherence (P): - MARS. Self-efficacy (P): - MASES. Schizophrenia symptoms (P): - PANSS.	4 months. Baseline, 3rd month.	1. ↑ Adherence in IG; ↓ adherence in CG over 3 months; and better adherence in IG in the 3rd month. Averaged MARS score increased from 7.2 (SD 1.9) at baseline to 7.4 (SD 1.9) in the 3rd month in IG. Averaged MARS score decreased from 6.9 (SD 1.9) at baseline to 6.8 (SD 2.2) in the 3rd month in CG. 2. Self-efficacy was unchanged over 3 months in both groups. 3. ↓ Schizophrenia symptoms in both groups over 3 months, and a greater reduction in symptoms was reported in IG in the 3rd month when compared to CG, Averaged PANSS scores decreased from 79.7 (SD 9.6) at baseline to 74.3 (SD 8.6) in the 3rd month in IG. Averaged PANSS scores decreased from 79.4 (SD 8.8) at baseline to 75.4 (SD 16.6) in the 3rd month in CG.
Beebe et al. 2017 [34]	Phone calls: Weekly phone calls with Telephone Intervention Problem Solving (TIPS) to address: (1) Knowledge of medication; (2) Attending appointments; (3) Coping with symptoms; (4) Abstaining from substances; (5) Social support. Guided participants through problem-solving, generating solutions, and following up on effectiveness.	TAU Usual monthly medication follow-up.	Medication adherence—pill counts in participants’ homes; Serum medication level tests.	6 months. Baseline, 6th month.	Pill counts No statistically significant difference in psychiatric medication adherence by pill count between groups at 6 months: 68.7% in IG vs. 63.9% in CG. Within IG: Adherence improved from 67.1% at baseline to 68.7% at 6 months, based on pill count. 2. Serum psychiatric medication levels A statistically significant difference (*p* = 0.023) was observed in serum antipsychotic medication levels within the therapeutic range at 6 months: 54.7% in IG vs. 32.7% in CG. Within IG: The percentage of participants with serum antipsychotic medication levels within the therapeutic range declined from 66% at baseline to 54.7% at 6 months; the significance of this change was not reported. For non-psychiatric medications, IG demonstrated higher adherence by pill count at baseline compared to CG (*p* = 0.02). However, no significant difference was observed between IG and CG after six months: 66.6% in IG vs. 71.63% in CG.
Uslu et al. 2020 [35]	Phone calls: Telephone Intervention Problem Solving (TIPS). Weekly telephone intervention by nurses to discuss problems and provide Medication Adherence Training (MAT).	TAU	Medication adherence (P): - MARS.	2 months. Baseline, 2nd month.	1. ↑ Adherence in IG, MARS median score (min–max) increased from 4 (3–6) to 9 (6–10) (*p* < 0.001); ↓ adherence in CG, MARS median score (min–max) decreased from 5 (1–8) to 4 (0–7) (*p* = 0.001). Effect between groups: MARS scores in IG were higher than in CG: Mann–Whitney U test: 4.5 (*p* < 0.001).
Frangou et al., 2005 [36]	Electronic medication monitors: @HOME Platform + MEMS: (1) A real-time remote monitoring platform using a medical sensor (MEMS); (2) Information is communicated to the clinical team daily; (3) Generates alerts; (4) Caregivers access the @HOME page.	1. TAU (Group A); 2. Pill counting (Group B).	Symptoms (P): - PANSS; - CGI-SCH. Medication adherence (P): - MAQ; - Pill count; - MEMS.	2 months. Baseline, 2nd month.	1. ↓ Symptoms in group B and IG in the 2nd month; greater improvement was reported in IG: PANSS total mean scores had significant differences among groups (F = 5.7, df = 2, *p* = 0.004). CGI total mean scores had significant differences among groups (F = 5.0, df = 2, *p* = 0.008). 2. Adherence in groups A and B was unchanged in the 2nd month; ↑ adherence in IG: IG showed a significant effect of group (F = 8.9, df = 2, *p* = 0.0001). Adherence in IG was significantly better than that in group A (*p* = 0.001) or group B (*p* = 0.007).
Kozuki et al., 2006 [37]	Electronic medication monitors: MEMS + Visual-feedback therapy, with behavioral and psychodynamic components; 6 sessions every two weeks. For behavioral components: electronic monitoring device, recorded daily medication. For psychodynamic components: participants express concerns and worries; the therapist interprets and discusses data from the electronic monitoring device.	Supportive counseling group	Medication adherence (P): - Electronic monitor (MEMS); - Blood plasma drug concentration; - Urine drug concentration; - Pill counts (For validation only). Symptoms (P): - PANSS.	3 months. MEMS: 2nd, 4th, 8th and 12th weeks. Plasma concentration: Baseline, 3rd month. PANSS: Baseline, 1st, 2nd, and 3rd months.	1. ↑ Adherence in IG over time; ↓ adherence in CG over time. Mean adherence rate in IG: 78.4%, 90.0%, 85.0%, and 87.9% at each time point, respectively. Mean adherence rate in CG: 88.5%, 82.3%, 75.9%, and 68.4% at each time point, respectively. Significant difference in adherence between IG and CG over time (F = 3.67, df = 3, *p* = 0.026). 2. Positive scores and negative scores showed no significant differences between the two groups: Positive scores: IG: 20.7 ± 2.6 (baseline) -> 20.0 ± 3.9 (3rd month); CG: 21.2 ± 4.3 (baseline) -> 23.0 ± 3.5 (3rd month). Negative scores: IG: 18.2 ± 4.1 (baseline) -> 17.9 ± 2.9 (3rd month); CG: 18.7 ± 3.3 (baseline) -> 19.7 ± 4.0 (3rd month).
Velligan et al., 2013 [38]	Electronic medication monitors: Med-eMonitor (MM) Prompting+ Phone call approach (Follow-up): (1) A smart pill container (Med-eMonitor TM); (2) Capable of cueing the taking of medication; (3) Warning patients when they are taking the wrong medication; (4) Recording side effect complaints; (5) Alerting treatment staff of failures to take medication.	1. PharmCAT (involves environmental modifications, such as healthcare providers placing medication reminder signs in patients’ homes). 2. TAU.	Medication adherence (P): - Electronic monitor (Med-eMonitor); - Pill counts. Symptoms (S): - BPRS. Global functioning (S): - SOFAS. Connect with emergency services: - Self-report on service utilization.	9 months. 3rd, 6th and 9th months. Pill count: Monthly.	1. Significant treatment group effect (F = 47.29, df = 2365, *p* < 0.0001) in IG. PharmCAT and IG were significantly better than TAU at all time points (*p* < 0.0001). The effect size for IG compared to TAU was 0.98 (i.e., a large treatment effect). 2. BPRS results showed no significant main effect or interaction (*p* > 0.09). 3. SOFAS scores showed no significant main effect or interaction (*p* > 0.09).

## Data Availability

No new data were created or analyzed in this study. Data sharing is not applicable to this article.

## Supplementary Materials

The following supporting information can be downloaded at: https://www.mdpi.com/article/10.3390/nursrep16090303/s1, File S1: PICO framework; File S2: Search strategies and history; File S3: Methodological quality of reviewed studies.

## Public Involvement Statement

There was no public involvement in any aspect of this research.

## Guidelines and Standards Statement

This review adheres to the Preferred Reporting Items for Systematic Reviews and Meta-Analyses (PRISMA) guidelines, ensuring structured and comprehensive reporting of the findings.

## Abbreviations

BARS	Brief Adherence Rating Scale
CAMH	Center for Addiction and Mental Health
CG	Control Group
CGI–SCH	Clinical Global Impression—Schizophrenia Scale
CGI–SCH-SI	Clinical Global Impression—Schizophrenia Scale, Severity of Illness
CGI–SI–DC	Clinical Global Impression—Schizophrenia Scale, Degree of Change
DAI-10	Drug Attitude Inventory—10
DSS	Social Disability Screening Schedule
EPHPP	Effective Public Health Practice Project
EQ-5D	The Spanish Version of the EuroQol
IG	Intervention Group
MAQ	Morisky Green Adherence Questionnaire
MARS	Medication Adherence Rating Scale
MASES	Medication Adherence Self-Efficacy Scale
MEMS	Medication Event Monitor System
PANSS	Positive and Negative Syndrome Scale
QLS	Quality-of-Life Scale
SOFAS	Social and Occupational Functioning Assessment Scale
SUMD	The Scale to Assess Unawareness of Mental Disorder
WHODAS	World Health Organization Disability Assessment Schedule 2.0.
