# Supplementary material for: Effectiveness of M-Health Interventions to Improve Medication Adherence in People with Schizophrenia Spectrum Disorder: A Systematic Review
Source: Nurs Rep. 2026 Aug 26;16(9):303. doi: 10.3390/nursrep16090303 (PMC13610701; doi:10.3390/nursrep16090303)
Supplement: Supplementary file 1 [file nursrep-16-00303-s001.zip › nursrep-4277473-supplementary.pdf]

Supplemental File S1: PICO framework

Participant (P)	Intervention (I)	Comparison (C)	Outcome (O)
Individuals with schizophrenia spectrum disorder	mHealth based intervention	Usual care	Medication adherence

Supplemental File S2: Search strategies and history

1. Search Strategies

1.1. PubMed

#1 schiz* [tiab]
#2 psychoses [tiab]
#3 psychosis [tiab]
#4 psychotic* [tiab]
#5 delusional* [tiab]
#6 #1 OR #2 OR #3 OR #4 OR #5
#7 mHealth [tiab]
#8 technolog* [tiab]
#9 app [tiab]
#10 application* [tiab]
#11 mobil* [tiab]
#12 smartphone* [tiab]
#13 phon* [tiab]
#14 tele* [tiab]
#15 text* [tiab]
#16 messag* [tiab]
#17 SMS [tiab]
#18 chat* [tiab]
#19 elec* [tiab]
#20 digi* [tiab]
#21 device* [tiab]
#22 monitor* [tiab]
#23 prompt* [tiab]
#24 reminder* [tiab]
#25 internet [tiab]
#26 wireless [tiab]
#27 web [tiab]
#28 website* [tiab]
#29 #7 OR #8 OR #9 OR #10 OR #11 OR #12 OR #13 OR #14 OR #15 OR #16 OR #17 OR #18 OR #19 OR #20 OR #21 OR #22 OR #23 OR #24 OR #25 OR #26 OR #27 OR #28
#30 adheren* [tiab]
#31 complian* [tiab]

#32 concordan* [tiab]
#33 allianc* [tiab]
#34 engag* [tiab]
#35 #30 OR #31 OR #32 OR #33 OR #34
#36 #6 AND #29 AND #35

1.2. CINAHL via EBSCO

S10 S4 and S9 Limiters - Full Text
S9 S5 or S6 or S7 or S8
S8 MW random* or TI random* or AB random* or IN random*
S7 MH triple-blind studies
S6 MH double-blind studies
S5 MH single-blind studies
S4 S1 and S2 and S3
S3 AB adheren* or AB complian* or AB concordan* or AB engag*
S2 AB mHealth or AB technolog* or AB app or AB application* or AB mobil* or AB smartphone* or AB phon* or AB tele* or AB text* or AB messag* or AB SMS or AB chat* or AB elec* or AB digi* or AB device* or AB monitor* or AB prompt* or AB reminder* or AB internet or AB wireless or AB web or AB website*
S1 AB schiz* or AB psychoses or AB psychosis or AB psychotic* or AB delusional*

1.3. PsycInfo via OvidSP

1. randomi$.mp.
2. ((singl$ or doubl$ or trebl$ or tripl$) adj (blind$ or mask$)).mp.
3. placebo$.mp.
4. exp placebo/
5. crossover.mp.
6. exp treatment effectiveness evaluation/
7. exp mental health program evaluation/
8. (random$ adj (assign$ or allocate$)).mp.
9. or/1-8
10. (schiz* or psychoses or delusional* or psychosis or psychotic*).mp.
11. (mHealth or technolog* or app or application* or mobil* or smartphone* or phon* or tele* or text* or messag* or SMS or chat* or elec* or digi* or device* or monitor* or prompt* or reminder* or internet or wireless or web or website*).mp.

12. (adheren* or complian* or concordan* or allianc* or engag*).mp.
13. 9 and 10 and 11 and 12
14. limit 13 to full text

1.4. EMBASE via OvidSP

1. (clin$ adj2 trial).mp.
2. ((singl$ or doubl$ or trebl$ or tripl$) adj (blind$ or mask$)).mp.
3. (random$ adj5 (assign$ or allocat$)).mp.
4. randomi$.mp.
5. crossover.mp.
6. exp randomized-controlled-trial/
7. exp double-blind-procedure/
8. exp crossover-procedure/
9. exp single-blind-procedure/
10. exp randomization/
11. 1 or 2 or 3 or 4 or 5 or 6 or 7 or 8 or 9 or 10
12. (schiz* or psychoses or delusional* or psychosis or psychotic*).mp.
13. (mHealth or technolog* or app or application* or mobil* or smartphone* or phon* or tele* or text* or messag* or SMS or chat* or elec* or digi* or device* or monitor* or prompt* or reminder* or internet or wireless or web or website*).mp.
14. (adheren* or complian* or concordan* or allianc* or engag*).mp.
15. 11 and 12 and 13 and 14
16. limit 15 to full text

1.5. JBI COnNect+ via OvidSP (Joanna Briggs Institute EBP Database)

1. (schiz* or psychoses or delusional* or psychosis or psychotic*).mp.
2. (mHealth or technolog* or app or application* or mobil* or smartphone* or phon* or tele* or text* or messag* or SMS or chat* or elec* or digi* or device* or monitor* or prompt* or reminder* or internet or wireless or web or website*).mp.
3. (adheren* or complian* or concordan* or allianc* or engag*).mp.
4. 1 and 2 and 3
5. limit 4 to full text

Supplementary File S3. Methodological quality of reviewed studies

Approach	Study	Selection Bias	Study Design	Confounder	Data Collection Method	Withdrawal and dropout	Global rating
**Text messaging interventions**	Montes et al., 2012	Weak	Strong	Strong	Strong	Strong	Moderate
	Xu et al., 2019	Strong	Strong	Strong	Strong	Strong	Strong
	Cullen et al., 2020	Moderate	Strong	Strong	Strong	Strong	Strong
	Cai et al., 2022	Strong	Strong	Strong	Strong	Strong	Strong
**Phone call approaches**	Beebe et al., 2008	Weak	Strong	Strong	Moderate	Strong	Moderate
	Beebe et al. 2016	Moderate	Strong	Weak	Strong	Weak	Weak
	Beebe et al. 2017	Weak	Strong	Weak	Strong	Weak	Weak
	Uslu & Buldukoglu., 2020	Moderate	Strong	Strong	Strong	Strong	Strong
**Electronic medication monitors**	Frangou et al., 2005	Moderate	Strong	Strong	Strong	Strong	Strong
	Kozuki & Schepp, 2006	Weak	Strong	Weak	Strong	Strong	Weak
	Velligan et al., 2013	Moderate	Strong	Strong	Strong	Strong	Strong
**Mobile applications**	Kidd et al., 2025	Moderate	Strong	Moderate	Strong	Moderate	Strong
	Liu et al., 2025	Moderate	Strong	Weak	Moderate	Strong	Moderate
	Zhou et al., 2025	Moderate	Moderate	Week	Moderate	Strong	Moderate
